# A SynNotch-PiggyBac gene circuit for inflammation-responsive IL-10 delivery in Alzheimer’s disease: a hypothesis and design framework

**DOI:** 10.3389/fnagi.2026.1866161

**Published:** 2026-10-09

**Authors:** Suhani Gupta, Ronav Gupta

**Affiliations:** 1 Neuro Health Alliance, Dublin, CA, United States; 2 IGNITE Pathways, Dublin, CA, United States

**Keywords:** AAV gene therapy, Alzheimer’s disease, astrocyte engineering, interleukin-10, neuroinflammation, PiggyBac transposon, SynNotch receptor, synthetic biology

## Abstract

Chronic neuroinflammation driven by sustained microglial and astrocyte activation contributes to Alzheimer’s disease progression, yet no therapeutic strategy addresses it with precision or durability in the brain. Interleukin-10 occupies an unresolved position in this problem. Constitutive interleukin-10 overexpression in amyloid precursor protein transgenic mice increased plaque burden by suppressing microglial phagocytic activity, while interleukin-10 deletion has been reported to restrict amyloidosis in one setting and to worsen tau hyperphosphorylation in another. We interpret this pattern as evidence that interleukin-10 carries opposite signs on the two principal pathologies of Alzheimer’s disease, so that any therapy holding it at a fixed concentration is committed in advance to being wrong about one of them. We therefore propose a falsifiable hypothesis: conditional, inflammation-gated interleukin-10 expression may suppress damaging inflammatory signaling while limiting the impairment of microglial phagocytosis associated with continuous exposure. We present a design framework for a synthetic gene circuit combining an adeno-associated viral vector for delivery to the central nervous system, a synthetic Notch receptor that detects membrane-bound tumor necrosis factor alpha on activated microglia through juxtacrine contact, and a PiggyBac transposon intended to integrate the circuit into the astrocyte genome. Receptor engagement is expected to release a transcription factor that drives interleukin-10 from a synthetic promoter, with transcription ceasing as the stimulus is withdrawn. We provide a quantitative packaging analysis showing that the circuit requires a compacted vector design to fit within viral capacity, and we identify three unvalidated assumptions: the recognition domain does not yet exist, ligand surface density on activated microglia is unmeasured, and shed soluble tumor necrosis factor alpha is predicted to act as a competitive inhibitor of receptor activation. A computational analysis of simulated resting-state electroencephalography, in which 45 of 171 electrode pairs differed after false discovery rate correction, is presented as a fully specified analysis workflow for delta-band connectivity as a secondary outcome measure rather than as evidence of effect, and yields effect sizes indicating that a rodent study would need roughly 11 animals per group. A four-arm validation plan is specified that separates the contribution of conditional regulation from that of genomic integration.

## 1 Introduction

Alzheimer’s disease (AD) is the leading cause of dementia in older adults, and an estimated 57.4 million people were living with dementia worldwide in 2019, a figure projected to reach 152.8 million by 2050 (GBD 2019 Dementia Forecasting Collaborators, 2022). The neuropathological hallmarks are extracellular amyloid-beta (Aβ) plaques and intraneuronal neurofibrillary tau tangles, and decades of drug development have concentrated on these two targets. The two most recently approved anti-amyloid antibodies, lecanemab and donanemab, slow cognitive decline in early-stage patients but do not halt progression (van Dyck et al., 2023), and neither addresses the sustained neuroinflammatory response that independently accelerates synaptic loss.

In the AD brain, microglia and reactive astrocytes remain chronically activated in the vicinity of amyloid plaques, releasing pro-inflammatory cytokines including tumor necrosis factor alpha (TNF-α) and interleukin-6 (IL-6) in self-amplifying cycles (Han et al., 2025). These cytokines promote tau hyperphosphorylation via p38 MAPK signaling, activate the NLRP3 inflammasome, compromise blood-brain barrier integrity, and damage synapses in ways that are not reversed by reducing amyloid burden alone (Mallick et al., 2025; Weston et al., 2021). Neuroinflammation therefore represents a mechanistically justified target in AD, and one that has proved difficult to address.

Attempts to address it have so far failed at the clinical stage, and the pattern of failure is informative. Non-steroidal anti-inflammatory drugs did not succeed in clinical trials, partly because systemic immune suppression introduces unacceptable infection risk in elderly patients without providing specificity to the central nervous system (CNS) (McGeer and McGeer, 2007). Biologic agents targeting individual cytokines face poor blood-brain barrier penetration at systemically safe doses. More recent efforts have narrowed the target without solving the underlying problem. A phase 2 trial of the TREM2 agonistic antibody AL002, designed to enhance microglial phagocytic function, achieved sustained target engagement in the CNS but did not meet its primary clinical endpoint (Mummery et al., 2026). A phase 3 program evaluating oral semaglutide moved biomarkers without delaying clinical progression (Cummings et al., 2026). What these attempts share is that each applies a fixed intervention to a process that varies over time and across brain regions.

Gene therapy delivering anti-inflammatory payloads directly into the brain offers a route to CNS specificity, and one attempt established a complication that has shaped the field since. Chakrabarty et al. (2015) used AAV2/1 to constitutively overexpress interleukin-10 (IL-10) in the brains of amyloid precursor protein (APP) transgenic mice and observed an increase in Aβ plaque burden, reduced microglial phagocytic activity, elevated apolipoprotein E in insoluble amyloid deposits, and worsened spatial memory. The mechanistic explanation was that sustained IL-10 signaling shifted microglia toward an immunosuppressive state that impaired clearance of Aβ. This result is widely read as evidence against IL-10 as a therapeutic target in AD.

We propose a different reading, and it rests on evidence that has accumulated on both sides of the question. Chakrabarty et al. (2015) demonstrated that continuous, unregulated IL-10 expression is harmful in an amyloid-forward model. Two loss-of-function studies complete the picture and point in opposite directions. Guillot-Sestier et al. (2015) reported that IL-10 deficiency in APP/PS1 mice revealed activated Aβ-phagocytic microglia, restricted amyloidosis, preserved synaptic integrity, and mitigated cognitive deficits. Weston et al. (2021) reported that IL-10-deficient mice challenged with systemic lipopolysaccharide showed greater tau hyperphosphorylation, sustained IL-6 elevation, and more amoeboid microglia. Read together, these results indicate that IL-10 carries opposite signs on amyloid and tau pathology. A therapy holding IL-10 at any fixed concentration is therefore committed in advance to being wrong about one of the two axes, which is a structural rather than a merely quantitative objection to constitutive delivery.

The hypothesis examined in this manuscript follows from that observation and is stated here in the form we intend to be tested: conditional, inflammation-gated IL-10 expression may suppress damaging inflammatory signaling while limiting the impairment of microglial phagocytosis associated with continuous IL-10 exposure. We propose a synthetic gene circuit in which astrocytes are engineered to produce IL-10 only in response to a detected inflammatory signal, using a synthetic Notch (SynNotch) receptor as the sensing and transduction mechanism. The receptor is designed to detect membrane-bound TNF-α on adjacent activated microglia through juxtacrine contact; on activation it undergoes proteolytic cleavage and releases a transcription factor that drives IL-10 expression. When the signal subsides, transcription is expected to stop. A PiggyBac transposon system delivered by adeno-associated virus (AAV) is proposed to integrate the circuit into the astrocyte genome, providing durability that episomal vectors may not sustain across the timescale of the disease.

The manuscript is organized to make the design testable rather than to advocate for it. Section 2 sets out the evidence base. Section 3 describes the circuit, and includes a quantitative packaging analysis and an examination of the sensing mechanism that identifies three assumptions on which the design depends and which are currently unvalidated. Section 4 presents a computational analysis of simulated electroencephalography (EEG) connectivity, offered as an illustrative workflow for a candidate outcome measure rather than as evidence of effect. Section 5 specifies a staged validation plan constructed to separate the two claims the design makes. Section 6 discusses novelty, limitations, and the experiments that could falsify the hypothesis.

## 2 Background and prior hypotheses

### 2.1 Neuroinflammation as a driver of Alzheimer’s disease

For much of the past three decades, neuroinflammation was treated as a secondary consequence of amyloid and tau accumulation rather than a primary driver of disease. Genome-wide association studies have substantially revised this view. Multiple AD risk loci map to genes expressed preferentially in microglia, including *TREM2*, *CD33*, and *BIN1*, indicating that innate immune dysregulation is embedded in the genetic architecture of the disease (Heneka et al., 2015). Epidemiological evidence shows that elevated systemic inflammatory markers in midlife predict AD risk decades later, and neuropathological studies document microglial and astrocyte activation preceding overt cognitive decline.

In the AD brain, activated microglia sustain release of TNF-α, IL-6, interleukin-1 beta, and reactive oxygen species (Han et al., 2025). Astrocytes adopt reactive states characterized by altered release of inflammatory mediators alongside changes in neuroprotective functions including glutamate reuptake and synaptic support. These states are heterogeneous and context-dependent rather than binary, and the consensus position of the field is that reactive astrocytes do not polarize into fixed neurotoxic and neuroprotective classes (Escartin et al., 2021). What the two glial populations establish jointly is a persistent cytokine milieu that promotes tau hyperphosphorylation through p38 MAPK signaling, activates the NLRP3 inflammasome, and disrupts synaptic communication (Weston et al., 2021). The resulting cycle, in which Aβ-driven inflammation damages neurons and neuronal damage amplifies glial activation, is not interrupted by any currently approved treatment.

### 2.2 IL-10 biology and the problem of fixed expression level

Interleukin-10 acts through the IL-10 receptor to activate JAK1 and TYK2, which phosphorylate STAT3 and suppress NF-κB-dependent inflammatory gene transcription (Lobo-Silva et al., 2016). Recent chromatin-level characterization in microglia has refined this account. IL-10 stimulation drives STAT3 occupancy at regulatory regions of *Il1b*, *Tnf*, *Il6*, and *Nlrp3*, with reduced RNA polymerase II and NF-κB binding at those loci, and repression involves a nuclear SHIP1-STAT3 complex with recruitment of histone deacetylases and reduced chromatin accessibility (Kim and Lee, 2026). In the healthy brain, IL-10 is produced by microglia and astrocytes following inflammatory stimuli and acts to terminate these episodes by reducing pro-inflammatory cytokine output and promoting a more homeostatic microglial state (Porro et al., 2020). In AD, this negative feedback mechanism is insufficient to resolve chronic activation.

Given that role, AAV-mediated IL-10 gene therapy appeared well reasoned. Chakrabarty et al. (2015) tested it directly. Neonatal TgCRND8 mice received intracerebroventricular injection of AAV2/1-IL-10 or AAV2/1-GFP control and were analyzed 6 months later. IL-10-expressing animals showed increased Aβ plaque burden by immunostaining and Thioflavin S staining, reduced microglial Aβ phagocytosis *in vitro*, elevated apolipoprotein E in insoluble amyloid fractions, and impaired performance on the Morris water maze. The mechanistic conclusion was that constitutive IL-10 chronically shifted microglia toward an immunosuppressive state that impaired phagocytic clearance of amyloid aggregates.

Two loss-of-function studies complete the picture, and they diverge. Guillot-Sestier et al. (2015), published in the same issue of *Neuron*, crossed APP/PS1 mice onto an *Il10*-null background. IL-10 deficiency revealed activated Aβ-phagocytic microglia, restricted cerebral amyloidosis, preserved synaptic integrity, and mitigated cognitive disturbance, with genome-wide RNA sequencing showing selective modulation of innate immune genes. Weston et al. (2021) examined the tau axis. *Il10*-deficient mice treated with intraperitoneal lipopolysaccharide showed markedly greater tau hyperphosphorylation at Ser396/Ser404 (PHF-1), AT180, and AT8 epitopes than wild-type controls, more amoeboid microglial morphology, and elevated brain IL-6 by multiplexed enzyme-linked immunosorbent assay (ELISA). Direct treatment of primary neurons with recombinant IL-6 reproduced phosphorylation at those same epitopes, establishing a mechanistic chain from IL-10 deficiency through IL-6 accumulation to tau pathology.

Read together, these three studies are not contradictory. Removing IL-10 improves amyloid outcomes and worsens tau outcomes; adding IL-10 continuously worsens amyloid outcomes. The consistent interpretation is that IL-10 carries opposite signs on the two principal pathologies of AD. A therapy holding IL-10 at any fixed concentration, whether elevated or absent, is therefore committed in advance to being wrong about one of the two axes. This is the observation on which the present proposal rests, and it is a stronger argument for conditional expression than a timing argument alone, because it identifies a structural rather than a quantitative problem with constitutive delivery.

Evidence from a related disease supports the therapeutic principle while leaving the AD question open. Bido et al. (2024) used a microRNA de-targeting system to achieve microglia-restricted IL-10 expression in a mouse model of Parkinson’s disease, and reported reduced neuropathology together with the emergence of microglial subtypes carrying enhanced clearance signatures. Cell-type-restricted IL-10 delivery to the CNS is therefore achievable and can be beneficial. What that work does not establish is whether the same holds in the presence of amyloid pathology, where the phagocytic burden on microglia is the central issue.

Two further considerations qualify how this reasoning should be applied. The first concerns the cellular target. IL-10 released near a plaque acts on microglia in a defined transcriptional state rather than on a homogeneous population. Plaque-associated microglia adopt the disease-associated phenotype, marked by downregulation of homeostatic genes including *Hexb*, *P2ry12*, and *Tmem119* and upregulation of *Trem2*, *Tyrobp*, *Apoe*, *Cst7*, and genes of lysosomal and lipid metabolism, with full induction dependent on TREM2 (Keren-Shaul et al., 2017; Deczkowska et al., 2018). Whether IL-10 opposes this program, is compatible with it, or reshapes it is unresolved, and it bears directly on whether conditional IL-10 preserves the phagocytic capacity the design assumes. We treat this as an open question and address it experimentally in Section 5.

The second concerns duration. Nothing in the evidence above establishes that brief IL-10 exposure is harmless to amyloid clearance. IL-10 produced during an inflammatory flare may reduce microglial Aβ uptake for the duration of that flare, and the therapeutic question is then whether the inflammatory benefit exceeds the clearance cost across repeated cycles. We state this explicitly because the design does not resolve it. The validation plan in Section 5 measures both quantities in the same animals rather than inferring one from the other.

### 2.3 SynNotch receptors as a platform for conditional gene expression

Synthetic Notch receptors were introduced by Morsut et al. (2016) from the Lim laboratory at the University of California San Francisco. They are modular chimeric proteins built on the core regulatory domain of human Notch1. In the native receptor, the extracellular domain binds Delta family ligands on adjacent cells, triggering sequential S2 cleavage by ADAM10 metalloproteases and S3 cleavage by the gamma-secretase complex, releasing the intracellular Notch domain for nuclear translocation. In the SynNotch architecture, both the extracellular recognition domain and the intracellular transcriptional domain are replaced with user-specified components, leaving the central regulatory region intact. The extracellular domain becomes a binder against a chosen surface antigen, and the intracellular domain becomes an orthogonal transcription factor driving a payload gene from a cognate synthetic promoter.

Because the SynNotch signaling pathway shares no intermediates with native Notch signaling, it does not activate endogenous Notch target genes, and multiple independent circuits can operate in the same cell without cross-talk (Morsut et al., 2016). The system requires direct cell-cell contact, functioning as a juxtacrine rather than a paracrine sensor. This property restricts payload production to cells in physical contact with the signal-presenting population, which concentrates the response at the anatomical site of interest.

Three properties of the platform constrain how it can be applied, and each is relevant to the design proposed here. Activation depends on ligand surface density relative to receptor affinity rather than on the mere presence of an antigen; a low-affinity HER2-directed SynNotch was induced by target cells displaying antigen on the order of 10^7^ molecules per cell but not by cells displaying approximately 6 × 10^4^ molecules per cell (Hernandez-Lopez et al., 2021). Activation is mechanically gated, requiring the ligand to be anchored so that binding generates tensile force across the negative regulatory region; soluble ligand fails to activate the receptor and acts as a competitive inhibitor (Sloas et al., 2023). Ligand-independent activation is a documented limitation, addressed in part by receptor variants incorporating an intracellular hydrophobic sequence from native Notch, which reduced tonic signaling by up to 14.6-fold without impairing antigen-induced activation (Yang et al., 2020).

Two experimental studies establish the relevance of the platform to the CNS. Liang et al. (2025) engineered brain astrocytes *in situ* with a dual-signal SynNotch system that detected two concurrent markers of neuronal apoptosis and released a neuroprotective factor in response, demonstrating that SynNotch circuits can be stably expressed and functionally responsive in CNS astrocytes under disease-relevant stimuli. Simic et al. (2024) used tissue-sensing T cells bearing a SynNotch circuit responsive to brevican, a CNS-specific extracellular matrix protein, to trigger local IL-10 production on CNS entry. This suppressed neuroinflammation in a murine experimental autoimmune encephalomyelitis model without causing systemic immunosuppression, validating SynNotch-driven, context-specific IL-10 delivery in a live animal.

A separate line of receptor engineering is relevant to translation. Systematic modular engineering of the SynNotch scaffold has produced synthetic intramembrane proteolysis receptors, which are more compact and fully humanized (Zhu et al., 2022). Because the transcription factor used in the original SynNotch design is derived from yeast and viral sequences, humanized scaffolds are the appropriate choice for any construct intended for sustained expression in human tissue, a point we return to in Section 3.2.

## 3 The SynNotch-PiggyBac IL-10 circuit: design and rationale

Table 1 places the proposed circuit alongside existing approaches to neuroinflammation in AD. No entry in its final column has been demonstrated for this circuit; the column describes design intent and the predictions that Section 5 is constructed to test.

**TABLE 1:** Comparison of neuroinflammation-targeted therapeutic approaches in Alzheimer’s disease.

Approach	Mechanism	Key limitations	Proposed advantages (not demonstrated)
NSAIDs and corticosteroids	Systemic cyclooxygenase inhibition; broad immune suppression	No CNS specificity; systemic immunosuppression; failed AD clinical trials (McGeer and McGeer, 2007)	Delivery is CNS-localized via AAV, so peripheral immune effects would not be expected
Anti-cytokine biologics	Neutralizing antibodies against TNF-α or IL-6	Poor blood-brain barrier penetration at safe systemic doses; disrupts normal cytokine signaling outside the CNS	Brain-targeted via AAV and an astrocyte-restricted promoter, avoiding peripheral cytokine blockade
Microglial activation therapies	TREM2 agonism to enhance phagocytic function	Target engagement achieved without clinical benefit in phase 2 (Mummery et al., 2026); amyloid-related imaging abnormalities observed	Modulates the inflammatory environment rather than forcing a single activation state
Constitutive IL-10 gene therapy	AAV-mediated continuous IL-10 overexpression in CNS cells	Increases amyloid burden by suppressing microglial phagocytosis (Chakrabarty et al., 2015)	Conditional expression is intended to limit IL-10 exposure to inflammatory episodes, on the hypothesis that phagocytic activity is preserved between them
SynNotch-PiggyBac circuit (proposed)	Feedback-regulated IL-10 secretion triggered by juxtacrine mTNF-α detection; integration via PiggyBac after AAV administration	Not experimentally validated; recognition domain not yet developed; soluble TNF-α predicted to act as competitive inhibitor; ligand-independent activation risk; insertional mutagenesis risk; dual-vector complexity	Expected to be self-regulating, durable after a single administration, and astrocyte-restricted, and to avoid constitutive immunosuppression

No entry in the final column has been experimentally demonstrated for the proposed circuit. Entries describe design intent and the predictions that Section 5 is constructed to test.

### 3.1 Astrocytes as the engineered chassis

The choice of cell type precedes the circuit design and should be justified independently of it. Astrocytes were selected for four reasons. They are abundant and long-lived, and adult parenchymal astrocytes are largely quiescent, which limits the clonal expansion that would amplify a mutagenic integration event in a proliferating population. They are in direct physical apposition to microglia at amyloid plaques, which is the spatial arrangement a juxtacrine sensor requires. They are efficiently transduced by several AAV serotypes and have well-characterized cell-type-restricted promoters available. And they are native producers of IL-10 following inflammatory stimulation, so the payload is not ectopic to the cell.

Recent work establishes that the chassis functions therapeutically in the AD brain. Chen et al. (2026) introduced anti-Aβ chimeric antigen receptors into astrocytes using AAV under a GFAP promoter, and reported that a single administration before plaque onset prevented deposition, while a single administration into animals with established pathology approximately halved plaque burden over 3 months, with single-nucleus RNA sequencing showing a coordinated astrocyte and microglial response.

Two alternative target populations deserve comment. Microglia present the ligand and might appear the more direct choice, but placing the circuit in them would convert a paracrine output arrangement into an autocrine loop, and IL-10 acting back on the cell that produced the triggering signal reproduces the sustained cell-autonomous suppression the design exists to avoid. Microglia are also inefficiently transduced by conventional AAV serotypes and they self-renew, which raises rather than lowers the risk associated with genomic integration. The microglia-restricted IL-10 approach of Bido et al. (2024) circumvented the delivery problem using lentiviral vectors with microRNA de-targeting, and succeeded in a Parkinson’s disease model where phagocytic clearance of amyloid was not the limiting consideration. Infiltrating immune cells engineered *ex vivo* are a validated route for CNS-directed IL-10 delivery (Simic et al., 2024), but that approach requires a per-patient cell product and does not persist without conditioning. The proposal here is for a single-administration vector therapy rather than a cell therapy.

### 3.2 Circuit architecture

The circuit consists of three linked molecular components intended to create a closed-loop, inflammation-responsive IL-10 expression system in engineered astrocytes. Figure 1 shows the architecture and both vector maps.

**FIGURE 1:**
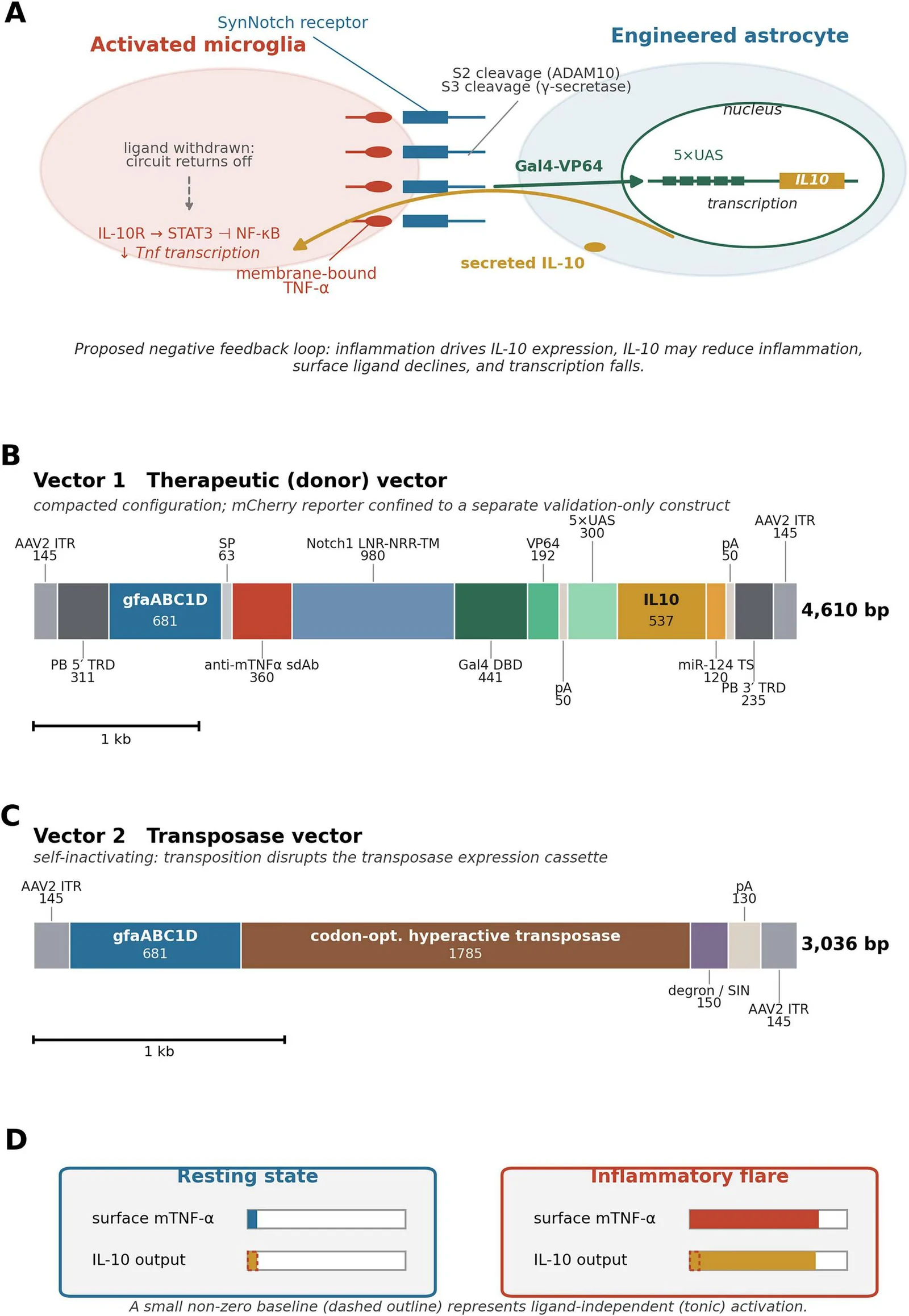
Proposed circuit architecture and vector design. **(A)** Circuit logic at the astrocyte-microglia interface. Membrane-bound TNF-α displayed on an activated microglial surface is engaged by the recognition domain of a SynNotch receptor expressed in an adjacent engineered astrocyte. Ligand binding under tensile load permits S2 cleavage by ADAM10 and S3 cleavage by gamma-secretase, releasing Gal4-VP64, which translocates to the nucleus and drives IL-10 transcription from a synthetic 5 × UAS promoter. Secreted IL-10 engages IL-10 receptors on the microglia, activating STAT3 and reducing NF-κB-dependent transcription of *Tnf*, which withdraws the ligand and closes the loop. **(B)** Therapeutic vector map drawn to scale, showing AAV inverted terminal repeats, PiggyBac 5′ and 3′ terminal repeat domains, the gfaABC1D promoter, receptor coding modules, the UAS-*IL10* cassette, and polyadenylation elements, annotated with the sizes given in Table 2. **(C)** Transposase vector map showing the self-inactivating configuration, in which transposition disrupts the transposase expression cassette. **(D)** Two-state representation of circuit behavior. In the resting state, membrane-bound TNF-α is at baseline and the UAS promoter is largely silent, shown with a small non-zero baseline that represents ligand-independent (tonic) activation. In the flare state, receptor engagement drives IL-10 output. No element of this figure has been experimentally validated.

The first component is the SynNotch receptor. The extracellular recognition domain is an antibody fragment engineered to bind membrane-bound TNF-α (mTNF-α) displayed on the surface of activated microglia. It is connected to the core Notch1 transmembrane region, which includes the negative regulatory region that prevents ligand-independent cleavage. The intracellular domain is the Gal4-VP64 synthetic transcription factor, comprising the Gal4 DNA-binding domain fused to the VP64 activation domain. The Gal4 domain has no known cognate binding sites in the human genome, so direct activation of endogenous genes is not expected.

The receptor scaffold requires a qualification that applies to translation rather than to proof of concept. Gal4 is derived from yeast and VP64 from herpes simplex virus, and chimeric transactivators of this kind have been reported to provoke immune responses that shorten transgene expression *in vivo*. Permanent genomic integration does not mitigate this and may compound it. We therefore propose Gal4-VP64 for initial work and a humanized synthetic intramembrane proteolysis receptor scaffold (Zhu et al., 2022) for any construct intended for translation, noting that humanized receptors drive weaker and slower payload expression, which is a trade-off requiring measurement rather than assertion. Receptor variants engineered to reduce ligand-independent activation (Yang et al., 2020) are proposed for both.

The second component is the IL-10 expression cassette. The *IL10* coding sequence is placed downstream of a synthetic promoter containing five Gal4 upstream activation sequence (UAS) repeats, which is expected to be silent in the absence of Gal4-VP64 and activated when the receptor releases it. In validation experiments an mCherry fluorescent reporter can be co-expressed via a T2A self-cleaving peptide to allow real-time visualization of circuit activation. As shown in Section 3.4, the reporter cannot be accommodated within the therapeutic vector and is therefore restricted to a separate validation construct.

The third component is the PiggyBac transposon system. Both the receptor gene and the UAS-*IL10* cassette are encoded within a transposon flanked by PiggyBac terminal repeat domains recognized by the PiggyBac transposase. After AAV delivery, the transposase catalyzes excision of the transposon from the episomal AAV genome and reinsertion into chromosomal TTAA sites. Once integrated, the circuit is expected to behave as a stable chromosomal element. The transposase is delivered by a second AAV in a self-inactivating configuration described in Section 3.6.

### 3.3 Sensor feasibility: membrane-bound TNF-α as a juxtacrine ligand

The choice of mTNF-α as the trigger carries most of the design risk, and three considerations qualify it. We set them out in full because the circuit’s viability depends on them and because each generates a decisive experiment.

The first is that no SynNotch receptor against membrane-bound TNF-α has been reported. Validated SynNotch recognition domains have been raised against CD19, HER2, GFP, ALPPL2, EGFRvIII, MCAM, EphA2, IL13Rα2, CD206, surface-displayed fluorescein, the CNS matrix protein brevican (Simic et al., 2024), and the phosphatidylserine and GT1b pair used in astrocytes (Liang et al., 2025). None is a shed cytokine. The recognition domain proposed here does not exist and would have to be developed and characterized before the circuit could be assembled. This is the largest single piece of unvalidated engineering in the proposal.

Epitope selection carries an additional constraint that follows from TNF biology. Membrane-bound TNF-α signals preferentially through TNFR2, which is associated with survival and neuroprotective outcomes, whereas the soluble form acts mainly through TNFR1 and drives the inflammatory program. Selective neutralization of soluble TNF-α while sparing the membrane-bound form is the basis of an ongoing clinical program in AD. A recognition domain intended as a sensor rather than as a neutralizer should therefore bind a surface distinct from the TNFR2 interaction interface, and confirming that receptor engagement does not impair TNFR2 signaling in the contacted microglia is a required control rather than an optional one.

The second consideration is ligand density. SynNotch activation depends on surface density relative to receptor affinity, and the available benchmark is not encouraging for a cytokine ligand: a low-affinity HER2-directed receptor was induced at approximately 10^7^ molecules per cell but not at approximately 6 × 10^4^ (Hernandez-Lopez et al., 2021). Surface densities for mTNF-α are unlikely to approach the upper figure, which places the burden on receptor affinity rather than on ligand abundance. Whether an affinity sufficient to close that gap can be achieved without also producing ligand-independent activation is an empirical question, and it is the first experiment specified in Section 5.1.

The third consideration concerns stability of the ligand, and it introduces a tension the design must confront directly. TNF-α is synthesized as a type II transmembrane protein and shed by ADAM17, whose activity rises within minutes of inflammatory stimulation. Cerebrospinal fluid ADAM17 activity is elevated in AD and in mild cognitive impairment relative to controls (Jiang et al., 2011). The inflammatory state the circuit is designed to detect is therefore also the state that most actively removes the ligand it detects. Because SynNotch activation is mechanically gated, the shed product is not merely unavailable but actively obstructive: soluble ligand occupies the binding site while providing no tensile resistance, and acts as a competitive inhibitor (Sloas et al., 2023). Shed soluble TNF-α would raise the effective activation threshold rather than being inert.

This property is not uniformly a liability. The same mechanical gating means that the circuit is not activated by cytokine acting at a distance, which is the specificity claim made for the design elsewhere in this section. Both consequences follow from the same mechanism and both are testable. Section 5.1 specifies a direct test: circuit output under ADAM17 inhibition should rise if surface retention of mTNF-α is the limiting variable, and a failure to observe this would indicate that the sensing premise is incorrect.

Spatial considerations favor the design. Microglia and astrocytes form reactive glial nets around compact amyloid plaques, astrocytic processes sprout into the plaque environment, and spatial transcriptomic characterization of the plaque niche describes direct microglia-astrocyte crosstalk with density-dependent effects on astrocyte state. Direct apposition of the two cell types at plaques is the normal condition rather than a rare event, which means the juxtacrine requirement functions as a spatial targeting mechanism concentrating circuit output where intervention is intended.

### 3.4 AAV delivery strategy and vector design

Adeno-associated virus is the established standard for clinical CNS gene delivery, with a safety record across multiple neurological trials (Ye et al., 2024). Two constraints shape the vector design: packaging capacity and the relationship between capsid tropism and promoter specificity.

#### 3.4.1 Packaging

Adeno-associated virus packaging capacity is approximately 4.7 kilobases, packaged genomes do not reliably exceed 5.0–5.2 kilobases regardless of construct or capsid, and titer falls sharply above 5.0 kilobases. Table 2 gives an element-by-element accounting for both vectors. The therapeutic vector as described in Section 3.2, including the mCherry-T2A reporter, totals approximately 5.84 kilobases between AAV inverted terminal repeats and would not package. Removing the reporter reduces this to approximately 5.06 kilobases, which sits at the practical ceiling and would be expected to yield low titer and a proportion of truncated genomes.

**TABLE 2:** Vector composition and packaging budget.

Vector 1: therapeutic (donor) vector	Size (bp)
AAV2 inverted terminal repeat (5′)	145
PiggyBac 5′ terminal repeat domain	311
gfaABC1D astrocyte promoter	681
Signal peptide	63
Anti-mTNF-α recognition domain (scFv)	750
Notch1 minimal regulatory region	980
Gal4 DNA-binding domain	441
VP64 activation domain	192
Synthetic polyadenylation signal (receptor)	150
5 × UAS with minimal promoter	300
Human *IL10* coding sequence	537
Synthetic polyadenylation signal (payload)	130
PiggyBac 3′ terminal repeat domain	235
AAV2 inverted terminal repeat (3′)	145
**Subtotal, no reporter**	**5,060**
T2A-mCherry reporter, if included	+780
**Total as originally conceived**	**5,840**
**Compacted configuration (proposed)**	**Saving (bp)**
Reporter confined to separate validation construct	−780
Single-domain antibody in place of scFv	−390
Synthetic minimal polyadenylation signals	−180
miR-124 target sequences added for neuronal de-targeting	+120
**Revised total**	**4,610**
**Vector 2: transposase vector**	**Size (bp)**
AAV2 inverted terminal repeats (both)	290
gfaABC1D astrocyte promoter	681
Codon-optimized hyperactive transposase	1,785
Degron and self-inactivating elements	150
Synthetic polyadenylation signal	130
**Total**	**3,036**

Sizes are representative values for commonly used components and require confirmation against final synthesized constructs. Practical AAV packaging capacity is approximately 4.7 kb; packaged genomes do not reliably exceed 5.0–5.2 kb and titer falls sharply above 5.0 kb. miR-124 target sequences for neuronal de-targeting are placed in the 3′ untranslated region and are itemized above.

We therefore propose a compacted configuration of approximately 4.61 kilobases, reached by three substitutions and one addition. The reporter is confined to a separate validation-only construct, consistent with its stated purpose. Conventional polyadenylation signals are replaced with synthetic minimal elements. The recognition domain is a single-domain antibody rather than a single-chain variable fragment, which reduces that module from approximately 750 to approximately 360 base pairs. The third substitution has a rationale independent of size, since a single-domain format simplifies the directed evolution required to tune affinity to the surface densities discussed in Section 3.3. miR-124 target sequences are then added to the 3′ untranslated region, at a cost of 120 base pairs, for the neuronal de-targeting described below. A compact humanized receptor scaffold would provide further headroom. The transposase vector totals approximately 3.04 kilobases and is not size-constrained.

#### 3.4.2 Capsid and promoter

The relevant property is not astrocyte transduction in isolation but the interaction between capsid tropism and promoter specificity, because cell-type-restricted promoters lose selectivity when paired with a capsid that has low affinity for the target cell. Among commonly used serotypes, AAV5 combined with the gfaABC1D promoter gives the highest reported astrocyte selectivity, and direct comparison of AAV5, AAV9, and AAVRec2 in spinal cord found AAV5 superior in transduction efficiency, transgene expression, and astrocyte tropism (Griffin et al., 2019). AAV9 and AAVrh10 are strongly neuron-selective in several comparisons, so pairing either with an astrocyte promoter is necessary but may not be sufficient. We therefore propose AAV5 with gfaABC1D. As a further restriction layer, incorporating miR-124 target sequences into the transgene 3′ untranslated region silences expression in neurons and has been shown to restore astrocyte specificity of gfaABC1D-driven expression where capsid tropism leaks (Taschenberger et al., 2017). These sequences are included in the design in Table 2, and the same de-targeting principle underlies the microglia-restricted IL-10 delivery of Bido et al. (2024).

#### 3.4.3 Species specificity

Serotype performance does not transfer across species, which constrains what preclinical results can be taken to show. AAV-PHP.eB, which achieves widespread CNS transduction after intravenous delivery in mice and was used in the chimeric antigen receptor astrocyte work (Chen et al., 2026), depends on LY6A, is restricted to particular mouse strains, and does not translate to non-human primates or humans. The gfaABC1D promoter has been characterized in rhesus macaque brain (Heffernan et al., 2022), so the promoter component has cross-species support that the capsid component does not. For initial proof-of-concept delivery in rodents, intracerebroventricular injection provides broad CNS distribution with minimal tissue trauma. Routes suitable for translation are discussed in Section 6.4.

### 3.5 Co-transduction requirements and the consequences of partial delivery

The dual-vector architecture requires that both genomes reach the same astrocyte, and this requirement deserves examination rather than assumption. Reported dual-AAV co-transduction efficiencies vary widely by tissue, capsid, and dose, with values around 5–10 percent relative to single-vector expression in some systems and substantially higher figures where capsid and dose are matched to the target population. No figure is available for astrocytes under the conditions proposed here, and establishing one is a prerequisite of the *in vivo* work.

Dose optimization is two-sided rather than monotonic. Insufficient transposase limits integration, while excess transposase is counterproductive through overproduction inhibition, so the ratio rather than the absolute dose of either vector is the operative variable. Hybrid viral and transposon delivery work indicates transposon to transposase ratios near 1000:1 as a starting point (Cooney et al., 2015).

The consequence of incomplete co-transduction is graded rather than binary, which bears on how much the design depends on this parameter. An astrocyte receiving only the therapeutic vector still expresses a functional conditional IL-10 circuit from the episome; it lacks permanent integration but is not therapeutically inert. An astrocyte receiving only the transposase vector expresses transposase without its cognate substrate, which is not necessarily inert, since the mammalian genome contains PiggyBac-derived sequences and remobilization of endogenous elements should be assessed rather than assumed absent. The population outcome of imperfect co-transduction is therefore a mixture of integrated and episomal circuits rather than a mixture of functional and non-functional cells. Partial delivery degrades durability rather than abolishing function, and the same reasoning weakens the case that integration is essential, which is why Section 5 tests that proposition rather than assuming it.

### 3.6 Self-regulating feedback logic

The circuit is designed to operate as a negative feedback loop governed by the local inflammatory state. When activated microglia expressing elevated mTNF-α contact an engineered astrocyte, the receptor binds mTNF-α, ADAM10 cleaves at the S2 site, and gamma-secretase cleaves at the S3 site, releasing Gal4-VP64. Gal4-VP64 enters the nucleus, binds UAS motifs upstream of the *IL10* gene, and recruits transcriptional machinery, leading to IL-10 production and secretion. Secreted IL-10 acts on IL-10 receptors on adjacent microglia, activating STAT3 and suppressing NF-κB-driven cytokine transcription, including transcription of *Tnf* itself (Kim and Lee, 2026). As microglial mTNF-α falls, the stimulus is withdrawn and IL-10 transcription would be expected to cease over a period of hours, a parameter that requires measurement rather than assumption.

This design is intended to address the expression timing problem identified by Chakrabarty et al. (2015). The hypothesis is that because IL-10 is produced only during active inflammatory episodes, microglial phagocytic activity is not suppressed during quiescent periods, and the amyloid clearance function that depends on maintained inflammatory competence is preserved between flares. We state this as a prediction rather than as a property of the design. Whether transient IL-10 during a flare reduces amyloid uptake for the duration of that flare, and whether the resulting trade-off is net favorable, are the questions Section 5.3 is constructed to answer.

Circuit sensitivity may be tuned by modifying the binding affinity of the recognition domain through directed evolution or rational mutagenesis. An AND-gate variant, in which two receptors targeting different inflammatory ligands must both be activated to drive expression, would increase specificity and reduce spurious activation, and this dual-input design has precedent in astrocytes (Liang et al., 2025). Both approaches interact with the ligand-independent activation problem discussed in Section 6.3, since increasing affinity to compensate for low ligand density also increases the likelihood of activation without ligand.

### 3.7 Genomic integration: rationale, alternatives, and safety

The case for integration rests on a narrower claim than durability alone. Adult parenchymal astrocytes are largely quiescent, so episomal dilution through cell division is not a substantial loss mechanism in this population, and the evidence on episomal persistence in post-mitotic CNS tissue is favorable. Tracking of AAV2 following striatal infusion in rats found transgene expression stable through 67 weeks (Gullapalli et al., 2026), transgene expression after intrastriatal AAV9 injection has been characterized over comparable periods (Hollidge et al., 2022), and the AAV2-BDNF phase 1 trial in AD has followed patients beyond 2 years with sustained metabolic effect in the treated region (Tuszynski et al., 2025).

The claim that remains is about extrapolation. AD requires intervention across decades rather than years, in aged tissue, under chronic inflammatory and oxidative stress, with epigenetic silencing of episomal genomes as a loss mechanism independent of cell division. Whether persistence measured over 2 years extends to twenty is unknown. We propose integration as a hedge against that uncertainty and, in Section 5, as a proposition to be tested rather than assumed.

Three alternatives merit comparison. Optimized episomal AAV with enhanced regulatory elements carries no insertional mutagenesis risk and improves expression stability, but does not address episomal loss itself; it is the appropriate control condition rather than a solution. Targeted integration at a defined safe-harbor locus using CRISPR-based methods is preferable in principle because the insertion site is specified rather than sampled, but homology-directed repair is inefficient in post-mitotic cells where non-homologous end joining predominates, nuclease, guide, donor and circuit together exceed AAV capacity by a wide margin even across two vectors, and persistent nuclease expression introduces a genotoxicity profile of its own. Non-integrating episomal delivery is the third alternative and is addressed by the comparator arm in Section 5. We regard targeted integration as the preferred long-term direction and PiggyBac as the more tractable option at present.

PiggyBac integrates at TTAA tetranucleotide sequences distributed throughout the genome with some preference for actively transcribed chromatin. This profile differs from retroviral vectors, which insert preferentially near transcriptional start sites and oncogene loci, but it is not without risk, and the following specifications are intended to constrain it.

#### 3.7.1 Transposase variant

A mouse codon-optimized hyperactive transposase, specified rather than left open. This is not a formality in neural tissue. Non-codon-optimized transposase introduced into developing mouse forebrain by in utero electroporation produced brain folding aberrations in all animals examined at embryonic day 18, while the codon-optimized variant produced a substantially milder and less frequent phenotype (Vierl et al., 2021). Excision-induced genomic alterations occur in approximately 0.8 percent of excision events with codon-optimized and hyperactive variants, below earlier reported footprint frequencies (Yusa et al., 2011).

#### 3.7.2 Self-limiting expression

The transposase coding sequence is positioned so that transposition disrupts its own expression cassette, making enzyme persistence inversely related to enzyme activity (Marh et al., 2012). A degron tag may be added to shorten protein half-life independently. Delivery of transposase as messenger RNA or as protein rather than as a vector-encoded gene would confine exposure to a defined window by construction and would remove the co-transduction requirement described in Section 3.5; neither format has a solved CNS delivery route, so both are discussed in Section 6.4 rather than proposed here.

#### 3.7.3 Copy number

A low integrated copy number per cell is the target, determined empirically by droplet digital polymerase chain reaction against a single-copy reference and by integration site sequencing. The trade-off is explicit: higher copy number increases expression level and durability while increasing insertional mutagenesis exposure, and the transposon to transposase ratio is the principal control variable.

#### 3.7.4 Remobilization

Integration site sequencing is performed at two time points separated by several months in the same animals, so that a change in the integration site profile would indicate continuing mobilization. The same analysis assesses whether transposase acts on endogenous PiggyBac-derived sequences.

#### 3.7.5 Long-term monitoring

We propose extended survival cohorts with histopathological examination for neoplasia, integration site profiling, and copy number stability. Rodent lifespan places a hard limit on what such monitoring can exclude for a therapy intended to act across human decades, and we state that limit in Section 6.3 rather than treating preclinical safety data as dispositive.

## 4 Computational analysis: an EEG connectivity workflow

### 4.1 Rationale

Neuroinflammation in AD disrupts brain network organization in ways that are measurable electrophysiologically. Cytokines released by chronically activated microglia and astrocytes alter glutamate receptor trafficking, reduce long-term potentiation, and impair the coordinated oscillatory activity across brain regions that underlies memory and executive function (Lecca et al., 2022). EEG studies of AD document elevated delta-band power and disruption of long-range frontoparietal coherence that correlates with cognitive severity (Cecchetti et al., 2024; Paitel et al., 2025).

Any *in vivo* evaluation of the proposed circuit will require a functional outcome measure sensitive to changes in neuroinflammatory burden, and delta-band connectivity is a candidate. The analysis below establishes and documents a processing and classification workflow for that purpose. It is presented as methodological groundwork for future experimental studies, and the limits of what it can support are set out in Section 4.4.

### 4.2 Methods

Simulated resting-state EEG data were generated for 24 sham (high-inflammation) and 24 treatment-simulated (lower-inflammation) participants using parameters derived from the published AD EEG literature. Analyses were implemented in Python 3. Signal processing, statistics, and classification are provided as a self-contained NumPy implementation, so that the analysis carries no dependency beyond NumPy and can be reproduced without matching a wider software stack. Each component was validated against published reference values rather than against the library it replaces: the Butterworth response is 0.7071 at both band edges and unity in the passband, the regularized incomplete beta reproduces standard tabulated values, the t survival function returns 0.05000 at published critical values, and the Benjamini-Hochberg routine reproduces the worked example of Benjamini and Hochberg (1995). Each participant’s data consisted of 30 s of 19-channel EEG at 256 Hz using the standard 10–20 electrode montage. Gaussian white noise with a standard deviation of 18 microvolts formed the background, and each participant received a distinct random seed so that no two simulated records are identical. Both conditions included a low-frequency drift component (4–14 microvolts at 0.05–0.2 Hz) to simulate physiological non-stationarity. The background noise and drift were generated identically in the two conditions, so that all systematic separation between them arises from the four features described next.

The sham condition received elevated delta-band oscillations (20–32 microvolts, 1.5–3.0 Hz) reflecting thalamocortical slowing documented in AD, weak posterior alpha activity (6–10 microvolts, 9–10.5 Hz), a strong shared 1.8 Hz component across occipitotemporal electrodes (15–22 microvolts) simulating aberrant local hyper-synchrony, and a weak shared 2.5 Hz component across frontoparietal electrodes (3–6 microvolts) representing long-range disconnection. The treatment-simulated condition received reduced delta amplitudes (9–16 microvolts), stronger posterior alpha activity (15–24 microvolts), attenuated occipitotemporal coupling (5–10 microvolts), and restored frontoparietal coupling (14–22 microvolts). Amplitudes and phases were drawn independently for each participant from these ranges. Complete parameter values, the per-participant seeds, and the generating code are available in the repository cited in the Data Availability Statement.

Each channel was bandpass filtered at 0.5–4.0 Hz with a fourth-order Butterworth filter applied forward and backward for zero phase distortion. Delta-band magnitude-squared coherence was then computed for every electrode pair by Welch’s method with 512-sample windows (2 s) and 50 percent overlap, and averaged across the 0.5–4.0 Hz bins. This produced a symmetric 19 by 19 matrix per participant whose upper triangle gives 171 unique electrode pairs. Differences between conditions were assessed by independent-samples *t*-tests for each pair, with false discovery rate control across all 171 tests by the Benjamini-Hochberg step-up procedure at q < 0.05.

Classification used the 171 coherence values as features. Within each fold, features were standardized to zero mean and unit variance, reduced to ten principal components, and passed to a support vector machine with a radial basis function kernel (*C* = 1.0, gamma set by the inverse of the product of feature count and feature variance) trained by sequential minimal optimization. Ten components were fixed in advance rather than tuned. Standardization and principal component analysis were fitted on the training partition only and then applied to the held-out observation, so that no information from a held-out participant informs its own transformation; fitting either step on the full dataset before splitting would leak information and inflate the estimate. Performance was evaluated by leave-one-out cross-validation across all 48 participants. Effect sizes were computed as Cohen’s d with pooled standard deviation for every pair reaching significance, and were used to derive the sample size a comparable rodent study would require, using a two-sided two-sample test at 80% power and an α = 0.05.

### 4.3 Results

Of the 171 electrode pairs tested, 45 showed differences in delta-band coherence between conditions surviving false discovery rate correction. The connectivity difference matrix (Figure 2A) shows predominantly positive values in frontoparietal electrode pairs where treatment-simulated coherence exceeded sham coherence, and negative values in temporal and occipital pairs. The network graph (Figure 2B) illustrates these connections. The dominant pattern of increased frontoparietal coupling and reduced occipitotemporal synchrony in the treatment-simulated condition corresponds to published EEG characterizations of AD network dysfunction (Cecchetti et al., 2024; Paitel et al., 2025), which is expected given that the simulation parameters were drawn from that literature.

**FIGURE 2:**
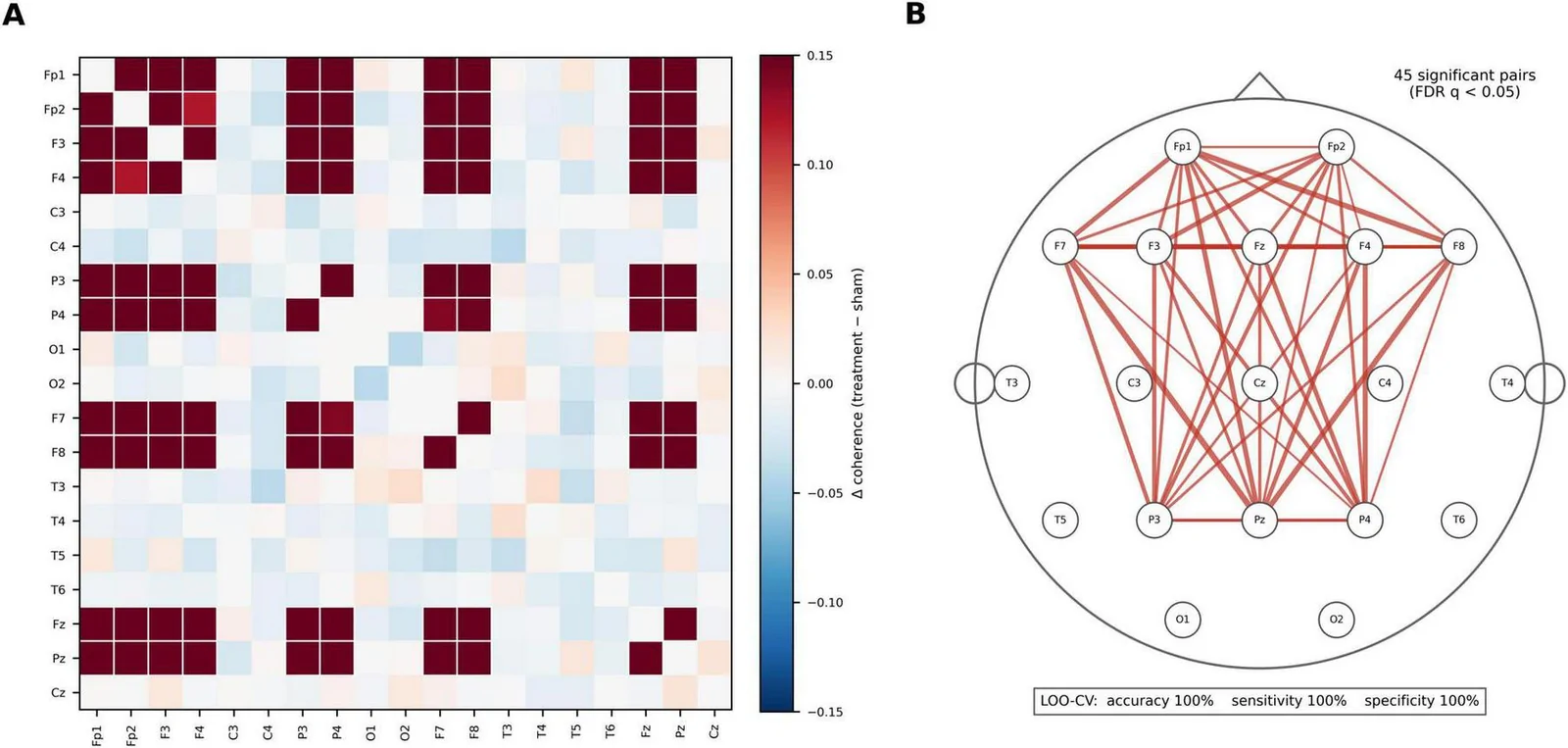
Delta-band (0.5–4 Hz) functional connectivity differences between simulated conditions (*n* = 48 simulated participants; 24 per group). **(A)** Heatmap of pairwise delta-band coherence differences across all 171 electrode pair combinations in the 19-channel standard 10–20 montage. Warm tones indicate higher coherence in the treatment-simulated condition; cool tones indicate lower coherence. White-bordered squares mark electrode pairs reaching significance after Benjamini-Hochberg false discovery rate correction (q < 0.05; 45 of 171 pairs). **(B)** Topographic network graph projecting significant differences onto a standard head schematic. Nodes represent electrode positions. Red edges indicate connections stronger in the treatment-simulated condition; blue edges indicate connections that are weaker. Edge width scales with effect magnitude. The inset reports classifier performance under leave-one-out cross-validation, with standardization and principal component analysis fitted within each fold on training data only. Complete separation is the expected consequence of simulating two conditions that differ systematically, and quantifies the ability of the pipeline to recover a difference specified when the data were generated. It is not a measurement of neuroinflammatory burden and does not bear on the efficacy of the proposed circuit.

Across the pairs reaching significance, the absolute effect size had a median of 2.80 and ranged from 1.26 to 5.52. These are large values, and they are large by construction rather than by discovery, since the connectivity difference between the two conditions was specified when the data were generated. Their practical use is in setting the sensitivity of a future experiment. An effect of the median simulated magnitude would be detectable with four animals per group at 80 percent power, while the smallest effect reaching significance here would require 11 per group. The latter figure is the appropriate basis for planning, and it is reported for that purpose in Section 5.3.

The classifier assigned all 48 participants correctly under leave-one-out cross-validation. Complete separation is the expected outcome and is not evidence of anything beyond the construction of the simulation. Two conditions that differ systematically in the amplitude of shared oscillatory components will produce coherence matrices that a classifier can separate, and the more faithfully the pipeline estimates coherence, the more completely it will do so. The result confirms that the workflow recovers a difference placed in the data; it says nothing about whether such a difference exists in a treated brain.

### 4.4 Interpretation of the computational analysis

The interpretation of this analysis must be stated narrowly. The two conditions were simulated with connectivity properties that were specified to differ, and the classifier recovered that difference. The classification accuracy therefore measures whether the analysis pipeline can detect a difference placed in the data by construction. It does not establish that neuroinflammation produces the reported network signature in a real brain, and it provides no evidence bearing on whether the proposed circuit would produce such a change. The simulation parameters were drawn from published descriptions of AD network dysfunction, which makes the simulated conditions plausible, but plausibility of the inputs does not convert a recovery result into a finding.

What the analysis provides is a specified and independently checkable workflow. Every processing decision, from filter order to the number of principal components, is fixed and documented, and the same pipeline is applied to the rodent recordings proposed as a secondary outcome in Section 5.3. Proposing an outcome measure without stating how it will be computed leaves a degree of analytical freedom that is difficult to audit after the fact. Fixing the pipeline in advance removes that freedom, and a simulation is a legitimate way to do so even though it cannot substitute for a measurement.

Two features of the analysis support this narrower reading. Standardization and dimensionality reduction were fitted inside each cross-validation fold rather than on the assembled dataset, so the accuracy is not inflated by information passing from held-out participants into their own transformations. Ten principal components were fixed before evaluation rather than selected to produce a particular figure. Both choices constrain the result downward, which is appropriate when the quantity of interest is whether a pipeline behaves sensibly rather than how well it can be made to perform.

The classification accuracy is therefore reported for completeness rather than as a finding, and we would discourage its citation as a performance figure. The quantities from this section that carry forward into the experimental plan are the effect sizes and the sample size derived from them.

The data are simulated and were not recorded from human participants or from any animal model. No experimental evidence of circuit efficacy is presented anywhere in this manuscript, and this section is not an exception.

## 5 Experimental validation plan

The plan is staged so that failure at an early stage prevents wasted effort at later ones, and it is constructed to separate the two claims the design makes. The first claim is that conditional regulation improves on constitutive delivery. The second is that genomic integration contributes durability beyond what episomal delivery provides. These are independent and are tested independently.

### 5.1 Stage 1: recognition domain development and sensor characterization

The circuit cannot be assembled until a recognition domain against mTNF-α exists. The first stage is its generation, by immunization or synthetic library selection, followed by affinity characterization by surface plasmon resonance and epitope mapping, with epitope selection constrained as described in Section 3.3 so that the binder does not occlude the TNFR2 interaction interface.

Six experiments then characterize the sensor.

1. Quantification of surface mTNF-α on resting and lipopolysaccharide-activated primary microglia by flow cytometry with quantitative bead standards, reported as molecules per cell so that the value can be compared against published activation thresholds.
2. Circuit activation as a function of mTNF-α surface density, performed both with microglia stimulated to graded levels and with a cell line displaying mTNF-α at defined densities, so that the dose-response relationship is established independently of microglial biology.
3. Direct comparison of membrane-bound and soluble TNF-α, including titration of recombinant soluble TNF-α against a fixed membrane-bound stimulus to determine whether it acts as a competitive inhibitor as predicted in Section 3.3.
4. Circuit activation under ADAM17 inhibition. If surface retention of mTNF-α is the limiting variable, reduced shedding should increase circuit output. Failure to observe this would indicate that the sensing premise is incorrect, and this experiment is included because it can falsify the design.
5. Confirmation that activation requires direct cell contact, using transwell separation and conditioned medium controls.
6. Assessment of activation by other mTNF-α-expressing populations, including peripheral macrophages, to establish the specificity boundary of the sensor.

Confirmation that receptor engagement does not impair TNFR2 signaling in the contacted microglia accompanies these experiments.

### 5.2 Stage 2: *in vitro* circuit validation in primary astrocytes

Primary cortical astrocytes derived from neonatal mouse cortex are transduced with the dual AAV system described in Section 3.4 and co-cultured with lipopolysaccharide-stimulated primary microglia. IL-10 secretion into culture medium is measured by ELISA at 24-h intervals over 72 h. The prediction is that IL-10 will be elevated in co-cultures containing activated microglia relative to unstimulated microglia co-cultures and to transduced astrocyte monocultures. The mCherry-T2A reporter construct allows fluorescence microscopy to quantify the fraction of transduced astrocytes in which the circuit is active and to confirm that activation requires direct contact.

Baseline IL-10 secretion from transduced astrocytes in monoculture, without stimulus, is measured throughout as the primary safety endpoint rather than as a specificity control. Ligand-independent activation is the failure mode that would reproduce the constitutive expression phenotype the circuit exists to avoid, and the acceptance criterion is that baseline IL-10 from unstimulated transduced astrocytes is indistinguishable from untransduced controls.

A non-integrating construct lacking PiggyBac terminal repeats is carried in parallel from this point onward, so that the contribution of conditional regulation is separable from the contribution of integration at every subsequent stage. Integration efficiency is assessed by genomic polymerase chain reaction spanning the terminal repeat junctions, with copy number by droplet digital polymerase chain reaction and chromosomal distribution by integration site sequencing, including proximity to annotated oncogenes and tumor suppressor loci. Long-term persistence is assessed by maintaining transduced astrocytes for 30 days without selection pressure and re-assaying IL-10 secretion after inflammatory stimulation, comparing the integrating and non-integrating constructs directly. Specificity controls include a non-binding recognition domain variant, co-culture with unstimulated microglia, and addition of soluble TNF-α without cell-cell contact.

### 5.3 Stage 3: *in vivo* validation

#### 5.3.1 Model selection

The critical contrast depends on measuring amyloid outcomes, which constrains the choice of model. Plaque appearance before 12 months in 3xTg-AD mice has become difficult to replicate in recent years, attributed to phenotypic drift on the mixed background, and the model shows large and inconsistent sexual dimorphism with colony source as a documented contributor to variability. A model in which amyloid pathology may be absent at the intended timepoint would place the central test at risk.

We therefore propose a two-model design. Amyloid-axis experiments use APP/PS1 mice, selected because this is the background on which IL-10 deletion was shown to restrict amyloidosis (Guillot-Sestier et al., 2015), which makes the result of the conditional circuit directly comparable to the strongest existing argument against IL-10 as a target. Tau-axis experiments use 3xTg-AD mice, which carry co-integrated human *APP*^Swe^ (KM670/671NL) and *MAPT*^P301L^ transgenes at a single locus under the mouse *Thy1* regulatory element, generated by pronuclear injection into embryos from homozygous *Psen1*^M146V^ knock-in mice, in which only the *Psen1* mutation is a targeted allele expressed from its endogenous locus (Oddo et al., 2003). Tau phosphorylation is read at AT180 and AT8 to connect directly to the epitopes reported by Weston et al. (2021). We expect the amyloid and tau results to dissociate, for the reasons given in Section 2.2, and state this in advance so that a partial result is interpretable rather than ambiguous.

#### 5.3.2 Study arms

Four arms are required. Vector control. Constitutive AAV-IL-10, reproducing the Chakrabarty et al. (2015) condition. Non-integrating conditional SynNotch-IL-10. Integrating SynNotch-PiggyBac-IL-10. The contrast between the second and third arms tests the timing hypothesis. The contrast between the third and fourth tests whether genomic integration contributes anything beyond conditional regulation. Without both contrasts neither claim can be attributed. Vectors are co-administered by intracerebroventricular injection at an age when neuroinflammation is developing but not yet maximal, set by the pathological timeline of each model rather than applied uniformly.

#### 5.3.3 Primary outcome: microglial phagocytic competence

Plaque burden alone would not establish whether microglial function was preserved, which is the mechanism the hypothesis concerns. Phagocytic capacity is therefore assessed directly, by *ex vivo* phagocytosis assay on acutely isolated microglia using fluorescent Aβ, by methoxy-X04 uptake *in vivo*, and by quantification of intracellular Aβ within IBA-1-positive cells in tissue sections. Plaque burden by Thioflavin S and 6E10 immunostaining is retained as a downstream consequence measure rather than as the test of the mechanism.

#### 5.3.4 Secondary outcomes

Brain IL-10 by ELISA and immunohistochemistry, with sampling designed to capture episodic rather than steady-state expression. Confirmation of integration and copy number. Neuroinflammatory markers by immunostaining for IBA-1 and GFAP and by western blot for TNF-α and IL-6. Tau phosphorylation at AT180 and AT8. Spatial memory by Morris water maze. Resting-state EEG analyzed by the delta-band connectivity workflow specified in Section 4, applied without modification so that the analysis is fixed before the recordings are made, with group sizes of at least 11 animals where this outcome is to be powered on its own.

#### 5.3.5 Transcriptional characterization

Single-nucleus RNA sequencing of treated tissue determines whether conditional IL-10 alters the disease-associated microglial program described in Section 2.2, and whether the glial response resembles or differs from that reported for astrocyte engineering by other approaches (Chen et al., 2026). This addresses the open question of whether IL-10 opposes or is compatible with the phagocytic program the design assumes it preserves.

#### 5.3.6 Safety

Integration site profiling at two time points, assessment of remobilization, extended survival cohorts with histopathological examination for neoplasia, and quantification of transposase persistence, as specified in Section 3.7.

## 6 Discussion

### 6.1 Novelty and contribution

The proposed circuit combines technologies validated independently, and the relationship to recent work should be stated precisely. Engineered astrocytes have now been shown to act therapeutically in the AD brain (Chen et al., 2026), which establishes the chassis and provides a benchmark for what single-administration astrocyte engineering achieves. The proposal here differs in what the engineered cell is made to do. Chimeric antigen receptor astrocytes are constitutive and act as effectors, clearing a substrate directly. The circuit proposed here is conditional and acts as an immunomodulator, releasing a secreted signal whose output tracks a state variable and ceases when that variable falls. The two approaches address different arms of the disease and are not alternatives to one another.

Synthetic Notch circuits have been applied in CNS astrocytes to detect markers of neuronal injury (Liang et al., 2025) and in immune cells to deliver IL-10 for suppression of CNS neuroinflammation in an animal model (Simic et al., 2024). Cell-type-restricted IL-10 gene delivery has been achieved in a Parkinson’s disease model using microRNA de-targeting (Bido et al., 2024). None of these combined conditional sensing with genomic persistence in astrocytes for AD. PiggyBac has been used in hybrid AAV delivery systems for non-neural gene therapy (Cunningham et al., 2015) and for stable expression in brain glia in developmental studies (Hamabe-Horiike et al., 2021), but not within a conditional feedback circuit.

The conceptual core of the contribution is the reinterpretation set out in Section 2.2, in which the divergent effects of IL-10 manipulation on amyloid and tau pathology are read as evidence that fixed-level delivery is structurally unsuited to the problem, together with a circuit design intended to test that reading and a validation plan constructed to separate its two claims.

The modularity of the architecture represents a broader advantage. The recognition domain, transcription factor, and payload can each be replaced independently, making the architecture adaptable to other neuroinflammatory conditions. A circuit delivering brain-derived neurotrophic factor in response to neuronal distress signals could address trophic loss in Parkinson’s disease; one delivering IL-10 in response to myelin-associated antigens could modulate inflammation in multiple sclerosis. The design principles apply across disease contexts at the architectural level.

### 6.2 Revisiting the constitutive IL-10 result

The observation that constitutive IL-10 increases amyloid burden (Chakrabarty et al., 2015) is mechanistically coherent. Sustained IL-10 signaling drives microglia toward a state characterized in part by reduced expression of complement receptor 3, which is required for efficient Aβ phagocytosis. Under chronic exposure, phagocytic capacity is reduced over prolonged periods, allowing amyloid to accumulate faster than it is cleared. This is a predictable homeostatic response to sustained cytokine signaling rather than an intrinsic property of IL-10 as a therapeutic molecule.

The proposed circuit is designed to make IL-10 signaling episodic rather than continuous. The hypothesis is that between inflammatory episodes, when mTNF-α is at baseline, the receptor is not engaged, engineered astrocytes release no IL-10, and microglial phagocytic and surveillance functions remain intact. This is the prediction the design exists to test rather than a property it possesses.

Two qualifications apply. Even transient IL-10 during a flare may reduce amyloid phagocytosis for the duration of that episode, and whether the resulting trade-off is net beneficial is unresolved; Section 5.3 measures both quantities in the same animals for this reason. And because IL-10 manipulation has opposite effects on amyloid and tau outcomes, the expected result of a successful experiment is not uniform improvement. A finding of reduced tau pathology and improved inflammatory markers with unchanged or modestly increased plaque burden would constitute informative partial confirmation rather than failure. We specify this interpretation in advance so that it cannot be applied retrospectively.

### 6.3 Limitations

Several limitations must be stated clearly. The circuit has not been built or tested. Every performance claim in this paper is a hypothesis derived from the component literature; no experimental evidence of circuit efficacy is presented.

The recognition domain does not exist. No SynNotch receptor against membrane-bound TNF-α has been reported, and the proposal depends on generating one with sufficient affinity to respond to a ligand whose surface density on activated microglia is unmeasured. This is the most consequential unvalidated element of the design.

Ligand availability may work against the design in the disease state. ADAM17-mediated shedding removes mTNF-α from the microglial surface, shedding activity is elevated in AD and mild cognitive impairment, and because SynNotch activation is mechanically gated the shed soluble form is predicted to act as a competitive inhibitor rather than as an inert byproduct.

Ligand-independent activation is the failure mode most likely to reproduce the phenotype the circuit exists to avoid. Tonic signaling is a documented limitation of SynNotch receptors, and increasing receptor affinity to compensate for low ligand density increases the risk. A circuit that leaks becomes a constitutive IL-10 vector with additional complexity.

The therapeutic vector is at or beyond AAV packaging capacity in the configuration originally conceived, and the compacted design in Section 3.4 has not been built. Whether the substitutions preserve function is unknown.

Insertional mutagenesis risk from PiggyBac integration, while reduced in largely quiescent astrocytes relative to proliferating cells, cannot be dismissed for a clinical application. Rodent lifespan places a limit on what preclinical monitoring can exclude for a therapy intended to act across human decades, and this limit is not removable by study design.

The dual-vector system introduces manufacturing and dose-ratio complexity, and co-transduction efficiency in astrocytes under the proposed conditions is unknown.

The juxtacrine requirement constrains the circuit to regions where engineered astrocytes and activated microglia are in direct contact. The spatial organization of the plaque niche makes this favorable in the primary target regions, but it remains a boundary condition.

Gal4-VP64 is derived from non-human sources and may provoke an immune response that shortens transgene expression, a risk that permanent integration does not mitigate.

The EEG analysis uses entirely simulated data. The classification accuracy reflects differences built into the simulation conditions and should not be interpreted as a clinical measurement or as evidence that the proposed circuit produces these effects.

### 6.4 Future directions

Transcriptomic characterization of astrocyte and microglial gene expression following circuit activation in co-culture would reveal whether induced IL-10 produces off-target effects on glial functional states. Induced pluripotent stem cell-derived human astrocytes, which more closely recapitulate the transcriptional identity of adult human CNS astrocytes, should be evaluated as a parallel model alongside mouse primary cultures.

A polycistronic payload co-expressing IL-10 and brain-derived neurotrophic factor within the same conditional circuit warrants investigation. AAV2-BDNF delivered to the entorhinal cortex of AD patients has been safe to date and restores metabolic activity in the treated region on FDG-PET (Tuszynski et al., 2025), supporting the relevance of trophic support as a complementary strategy. Combining anti-inflammatory and neurotrophic payloads within one conditional circuit would address two independent drivers of neurodegeneration while maintaining conditional expression logic.

For delivery, intracerebroventricular injection is appropriate for rodent proof of concept but is not viable at population scale. Intravenous administration using capsids engineered to cross the blood-brain barrier is the translational path, and the relevant candidates have changed. AAV-PHP.eB does not translate beyond particular mouse strains, whereas capsids engineered to bind human transferrin receptor 1 are designed for cross-species function and are entering clinical development for neurological indications. Focused ultrasound-mediated blood-brain barrier opening combined with intravenous delivery offers a route to spatially targeted transduction without cranial surgery and is compatible with the dual-vector approach.

Two changes to the integration strategy would reduce its risk profile. Delivering the transposase as messenger RNA or as protein rather than as a vector-encoded gene would confine exposure to a defined window by construction and would remove the co-transduction requirement described in Section 3.5, with CNS delivery of either format the obstacle. Targeted integration at a defined safe-harbor locus would replace semi-random insertion with a specified one, and the obstacles described in Section 3.7 are engineering problems rather than problems of principle.

## 7 Conclusion

We have presented a hypothesis and design framework for a conditional gene circuit addressing the neuroinflammatory axis of Alzheimer’s disease, an axis mechanistically established as a driver of pathology but therapeutically underaddressed. The central argument is that IL-10 has opposite effects on amyloid and tau pathology, so the failure of prior IL-10 gene therapy reflected the structural unsuitability of fixed-level delivery rather than an error in target selection. A SynNotch receptor-based feedback system integrated into astrocytes following AAV delivery may produce IL-10 during neuroinflammatory flares while remaining inactive between them, and thereby preserve the microglial phagocytic activity that constitutive overexpression compromised.

The design builds on foundations that have been validated separately: SynNotch receptors that function in CNS astrocytes and can drive context-specific IL-10 delivery, PiggyBac-mediated integration via hybrid AAV systems, AAV-based CNS gene therapy with an established clinical safety record, and the demonstration that AAV-engineered astrocytes act therapeutically in the AD brain. Their combination into a conditional, self-regulating astrocyte platform for AD neuroinflammation has not been described previously.

The analysis presented here has also identified constraints on the design that were not evident at the outset. The recognition domain does not exist. The vector as first conceived exceeds packaging capacity and requires the compacted configuration specified in Section 3.4. Enhanced shedding of the target ligand in the disease state is predicted to raise rather than lower the activation threshold. We report these because a hypothesis is more useful when the conditions under which it would fail are stated alongside the conditions under which it would succeed.

The computational EEG analysis is presented as an illustrative analysis workflow and a design basis for a candidate secondary outcome measure. It does not support the proposal that the circuit produces connectivity changes and is not offered as evidence of therapeutic effect.

This work is a hypothesis requiring experimental validation, and that must be stated plainly. Its value lies in providing a theoretically grounded and technologically specified design that is directly testable in defined preclinical systems, together with a validation plan constructed so that its two claims can be separated and either can be falsified.

## Data Availability

The simulated EEG datasets, the complete simulation parameter values, the per-participant random seeds and the analysis code supporting the conclusions of this article are publicly available at https://github.com/suhanigupta3/SynNotch-PiggyBac-Gene-Circuit-Data.
